# Case report: difficulty in diagnosis of delayed spinal epidural hematoma in puerperal women after combined spinal epidural anaesthesia

**DOI:** 10.1186/s12871-019-0721-y

**Published:** 2019-04-11

**Authors:** Alessandro Svelato, Alberto Rutili, Caterina Bertelloni, Domenico Foti, Angela Capizzi, Antonio Ragusa

**Affiliations:** 1 Department of Obstetrics and Gynecology, Massa Carrara General Hospital, Via Enrico Mattei, 54100 Massa Carrara, Italy; 2 Department of Anaesthesia, Massa Carrara General Hospital, Massa Carrara, Italy; 3 Department of Neurosurgery, S. Chiara Hospital, Pisa, Italy; 4 Department of Obstetrics and Gynecology, Imperia General Hospital, Imperia, Italy

**Keywords:** Spinal epidural hematoma, Obstetric, Labour, Epidural, Diagnosis, Case report

## Abstract

**Background:**

Spinal epidural hematoma is a rare but serious complication of epidural anaesthesia and neurological impairment. Epidural hematoma usually becomes evident within a few hours of the procedure. Delayed clinical presentation of spinal epidural hematoma is even rarer and insidious.

**Case presentation:**

We reported a case of a 44-year-old woman who underwent a caesarean section for a twin pregnancy during which a delayed dorsal spinal epidural hematoma occurred. Symptoms were reported 5 days after surgery and 72 h after removal of the epidural catheter. An MRI scan showed a dorsal epidural hematoma. The patient was moved to the Neurosurgical Department and underwent decompression surgery.

**Conclusion:**

The possibility of the delayed onset of a spinal epidural hematoma in a pregnant woman who undergoes epidural anaesthesia in labour must always be taken into consideration. In order to achieve the best clinical result, we stress the importance of a timely diagnosis and prompt surgical treatment.

## Background

Spinal Epidural Hematoma (SEH), a rare but potentially devastating complication, is a symptomatic bleeding within the spine where blood accumulates outside the dura, mostly caused by traumatic or iatrogenic (spinal surgeries, obstetrical birth trauma, lumbar puncture, spinal manipulations, and epidural procedures) events [1]. SEH can cause rare but potentially catastrophic compression of neural tissue by direct injury or ischemia, with an incidence ranging from 1:2600 to 1:220.000 [2–6]. Parturients can also be predisposed to the development of an epidural hematoma, due to a decreased number or function of platelets (i.e., with pre-eclampsia or hemolysis, elevated liver enzymes, low platelet count [HELLP] syndrome) or anticoagulant use, even if pregnancy is a prothrombotic, hypercoagulable state [2].

In this manuscript we report a case of difficult diagnosis concerning a delayed epidural hematoma, due to combined spinal epidural (CSE) anaesthesia in a caesarean delivery.

## Case presentation

A 44 year-old pregnant nulliparous, weight 70 Kg, height 172 cm, BMI 23.7, at 34 weeks of a twin gestation, obtained by “in vitro” fertilization, was admitted to the General Hospital of Massa, complaining spreading pricking and lower limb edema. The patient suffered from unstable insulin-dependent type I diabetes and sciatica. Considering 4 days of immobilization in bed, low molecular weight heparin (Dalteparin© 2500 UI one per day) was administered [7]. The patient underwent a planned caesarean section under double-space CSE anaesthesia, using a 25-gauge atraumatic spinal needle at level L3-L4 and an 18-gauge Thuoy needle at T12-L1 (B. Braun Perifix® epidural set), placed during a single attempt. Preoperative coagulation parameters were within normal range (Platelet count 120.000 per microliter of blood; Prothrombin time 12 s, Partial thromboplastin time 30 s, fibrinogen 540 mg/dL, INR 0.90). Renal function was normal. The operation was carried out routinely and multimodal pain therapy was started (Patient Controlled Epidural Anaesthesia - PCEA - with chirocaine 0,15% plus sufentanil 0,5 mcg/ml, 4 ml/h; i.v. ketorolac 30 mg/day and oral tramadol 30 mg plus acetaminophen 1000 mg/3 times/day). Four hours after the operation, the urinary catheter was removed, the patient began to stand up and to take care of the newborns. Dalteparin 2500 U/die was continued. PCEA was discontinued 2 days after the operation and the epidural catheter was removed on the 3rd day, 12 h after the last Dalteparin administration. Pain control was optimal. The following day, the patient remained hospitalized without any complications and the two babies were admitted in neonatology.

**Fig. 1:**
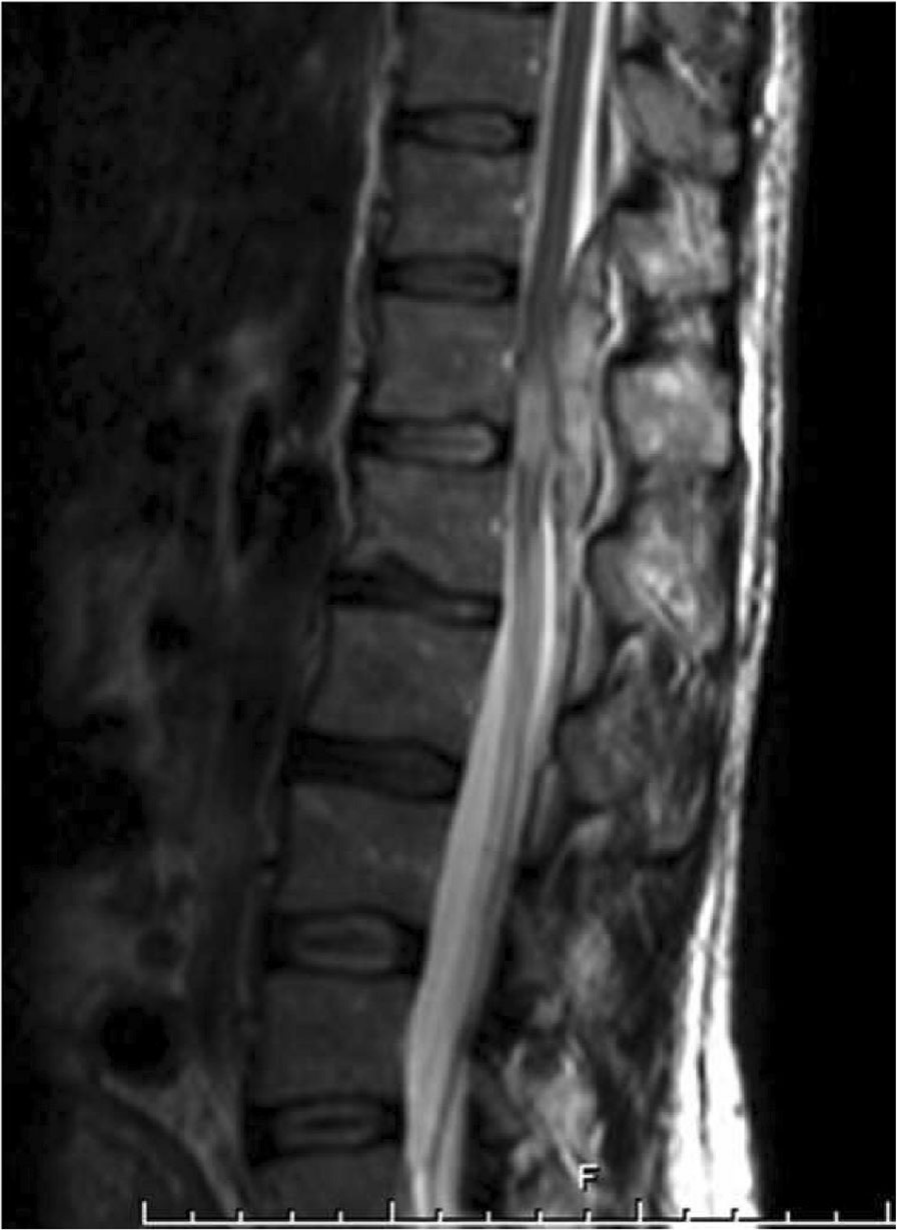
Sagittal T2-weighted MRI shows hyperintense collection in the posterior spinal epidural space at level D12-L1 and spinal cord signal alteration

**Fig. 2:**
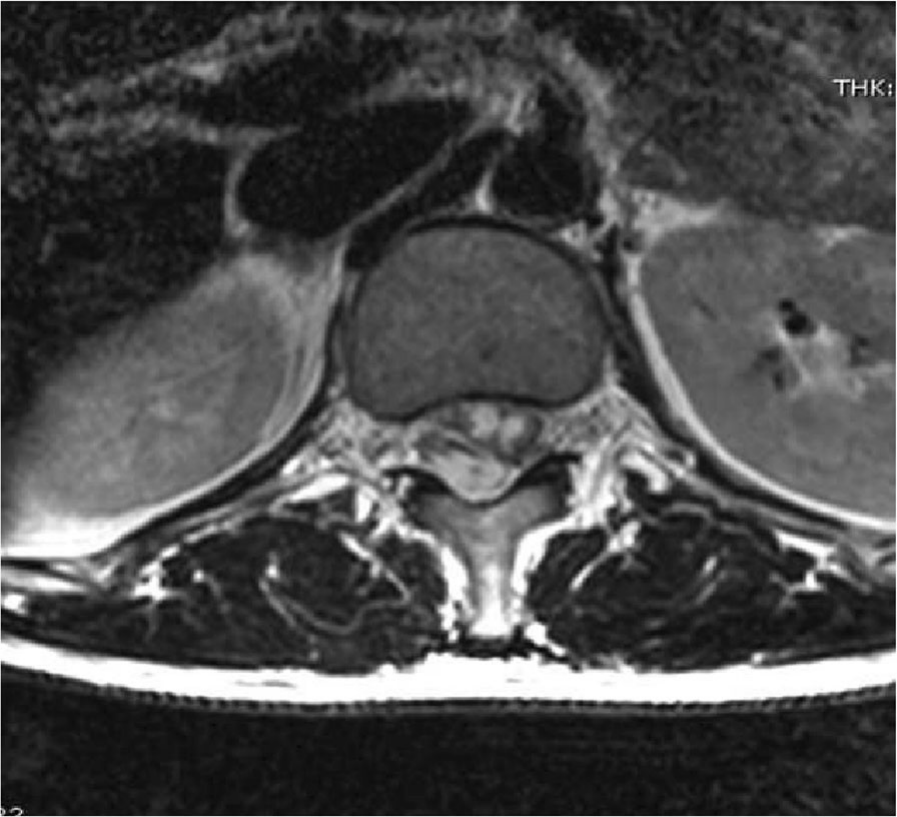
Axial T2-weighted MRI shows hematoma compressing spinal cord from the right side

At 06.00 a.m. of the 6th post-operative day, more than 70 h after the removal of the epidural catheter, the patient complained of acute and severe low-back pain, radiating to the right inferior limb, and paresthesia; there was no motor impairment and she was treated with analgesic drugs without any improvement. After an abdominal ultrasound, urinary retention was observed and a bladder catheter was inserted (residual volume: 1100 ml). A right lower limb motor deficit was observed at 11:50 a.m. and methylprednisolone was administered. Since no improvement in the patient’s clinical picture was seen, urgent neurological consultation was requested 8 h after the onset of symptoms (02.00 p.m.). A dorsal MRI scan showed the presence of a T12-L1 posterior SEH, predominantly on the right side, with significant mass-effect and spinal cord signal alteration in the conus medullaris region (Figs. 1 and 2). The patient was transferred to the Neurosurgical Department for decompression laminectomy and removal of the hematoma and the operation began at 07.00 p.m.. Immediately after surgery, the patient partially recovered her sensory disorder and motor functions. Five days after surgery she began intensive rehabilitation.

In the last follow-up examination (36 months later), while mostly improved, motor, sensory and sphincter deficits persisted in varying degrees. The last MRI confirmed signs of permanent ischemic injury at level T12-L1.

## Discussion and conclusion

Neuraxial techniques are used to provide analgesia for labour [8, 9] and anaesthesia for surgical delivery. Therefore, complications associated with neuraxial techniques are sometimes seen even in pregnant patients.

We have described a case of SEH, one of the more severe complications.

In our case, the acute and severe low-back pain, radiating to the right inferior limb, and paresthesia were the first signs reported by the patient. The physicians focused their attention only on the medical history of the patient’s sciatica, without first ruling out the more dangerous possible diagnosis, even if less frequent. Moreover, they also ignored the risk factors present. There are many risk factors for SEH, but is know that the most important are spinal/epidural procedures in combination with anticoagulant use [2, 3]. We used a double-space CSE anaesthesia, because a caesarean section is a major surgery, along with the concerns of potential surgical complications and longer operating time. If an epidural catheter is available, our protocol for postoperative pain management considers the possibility to prolong epidural analgesia for 48 h, in line with SIAARTI guidelines [10–12]. Moreover, our hospital is a Baby Friendly Hospital, which means that is at the forefront in the promotion of breastfeeding. In this view, postoperative pain control is of primary importance, with the aim of promoting skin to skin contact and the start of breastfeeding. Anticoagulation is also a risk factor for SEH, however the doses of drugs with effects on coagulation that we used, were very low (Deltaparina 2500 UI once daily and ketorolac 30 mg/die for 2 days) in consideration of the patient’s weight (70 Kg) [7].

A delay in diagnostic imaging can lead to devastating outcomes, and is an error, since neurological symptoms and back pain can be attributed to epidural infusion and a prolonged effect of the local anaesthetic, or to a musculoskeletal origin [13].

Another element that made the diagnosis difficult was the appearance of the symptoms 3 days after the removal of the epidural catheter. This lag time is possible [14].

The second sign was urinary retention, followed by motor impairment. This case evolution is typical of a SEH [1]. In 30% of cases there is an acute onset of complete paralysis with bowel or bladder disturbances, while in 16% of cases, as well as in our case, the onset is less typical with incomplete paralysis [1, 3]. The presence of motor deficit is an indication for surgery [15]. In the case of sensory symptoms without motor deficit, surgery does not seem to improve outcomes and a wait-and-see approach, with meticulous follow up, seems to be the best option [15]. In the case of motor deficit, as well as in our case, decompression laminectomy within 12 h results in the best surgical outcome [3, 15, 16]. Nevertheless, literature reports cases of good outcome also in the case of surgery performed after 24 h from the onset of symptoms [15].

In conclusion, we should always suspect a delayed onset of SEH in a patient who undergoes epidural anaesthesia or other similar neuraxial procedures. We stress the importance of an early diagnosis of this serious complication and of prompt surgical treatment to maximize neurological improvement. Table 1 lists the most common “red flags” to notice and pitfalls to avoid for a prompt diagnosis.

**Table 1:** Red flags and pitfalls in the diagnosis of spinal epidural hematoma

Red flags in the diagnosis of spinal epidural hematoma	
1) Considering only the most frequent diagnosis, without excluding serious but infrequent diagnoses	
2) Persistent motor block	
3) Bowel/bladder dysfunction	
4) Other symptom (usually back or leg pain)	
Pitfalls in the diagnosis of spinal epidural hematoma	
1) Ignoring the risk factors	
2) Administering treatment before making the diagnosis	
3) Not performing surgery only because too much time has passed since the appearance of the symptoms (> 12 h)	

After this case we performed an audit that involved all staff members and we have introduced some actions of improvement. For example, we have changed the number of patient evaluation from 1 per day to 2 per day. We have organized a review day about SEH with the aim to inform all staff members about the onset of symptoms and early diagnosis of this condition.

The most important lesson to be learned from our case is thinking that SEH exists and is a potential complication of epidural anaesthesia, so we must always take this complication into consideration in the case of neurological symptoms appearance.

Significant back pain and/or lower limb pain, even if nonspecific symptoms, and in the absence of motor impairment, should alert all staff members to request an urgent neurological, neuroradiological and neurosurgical consultation, even in the late stages of patient hospitalization.

## Abbreviations

CSE: Combined spinal epidural
PCEA: Patient Controlled Epidural Anaesthesia
SEH: Spinal Epidural Hematoma

## References

[1] Shaban A, Moritani T, Al Kasab S, Sheharyar A, Limaye KS, Adams HP (2018). Spinal cord hemorrhage. J Stroke Cerebrovasc Dis.

[2] Hoefnagel A, Yu A, Kaminski A (2016). Anesthetic complications in pregnancy. Crit Care Clin.

[3] Kreppel D, Antoniadis G, Seeling W (2003). Spinal hematoma: a literature survey with meta-analysis of 613 patients. Neurosurg Rev.

[4] Bateman BT, Mhyre JM, Ehrenfeld J, Kheterpal S, Abbey KR, Argalious M, Berman MF, Jacques PS, Levy W, Loeb RG, Paganelli W, Smith KW, Wethington KL, Wax D, Pace NL, Tremper K, Sandberg WS (2013). The risk and outcome of epidural hematomas after perioperative and obstetric epidural catheterization: a report from the multicenter perioperative outcomes group research consortium. Anesth Analg.

[5] D’Angelo R, Smiley RM, Riley ET, Segal S (2014). Serious complications related to obstetric anesthesia: the serious complication repository project of the Society for Obstetric Anesthesia and Perinatology. Anesthesiology.

[6] Ruppen W, Derry S, McQuay H, Moore RA (2006). Incidence of epidural hematoma, infection, and neurologic injury in obstetric patients with epidural analgesia/anesthesia. Anesthesiology.

[7] Leffert Lisa, Butwick Alexander, Carvalho Brendan, Arendt Katherine, Bates Shannon M., Friedman Alex, Horlocker Terese, Houle Timothy, Landau Ruth, Dubois Heloise, Fernando Roshan, Houle Tim, Kopp Sandra, Montgomery Douglas, Pellegrini Joseph, Smiley Richard, Toledo Paloma (2018). The Society for Obstetric Anesthesia and Perinatology Consensus Statement on the Anesthetic Management of Pregnant and Postpartum Women Receiving Thromboprophylaxis or Higher Dose Anticoagulants. Anesthesia & Analgesia.

[8] Ragusa Antonio, Gizzo Salvatore, Noventa Marco, Ferrazzi Enrico, Deiana Sara, Svelato Alessandro (2016). Prevention of primary caesarean delivery: comprehensive management of dystocia in nulliparous patients at term. Archives of Gynecology and Obstetrics.

[9] Svelato A, Di Tommaso M, Spinoso R, Ragusa A (2016). The reduction of first cesarean sections: a cultural issue. Acta Obstet Gynecol Scand.

[10] Gogarten W, Vandermeulen E, Van Aken H, Kozek S, Llau JV, Samama CM (2010). Regional anaesthesia and antithrombotic agents: recommendations of the European Society of Anaesthesiology. Eur J Anaesthesiol.

[11] Bertini L, Savoia G, De Nicola A, Ivani G, Gravino E, Albani A, Alemanno F, Barbati A, Borghi B, Borrometi F, Casati A, Celleno D, Ciaschi A, Corcione A, De Negri P, Di Benedetto P, Evangelista M, Fanelli G, Grossi P, Loreto M, Margaria E, Mastronardi P, Mattia C, Nicosia F, Nolli M, Rutili A, Santangelo E, Sucre J, Tagariello V, Varrassi G, Paoletti F, Tufano R (2006). SIAARTI. SIAARTI guidelines for safety in locoregional anaesthesia. Minerva Anestesiol.

[12] Savoia G, Alampi D, Amantea B, Ambrosio F, Arcioni R, Berti M, Bettelli G, Bertini L, Bosco M, Casati A, Castelletti I, Carassiti M, Coluzzi F, Costantini A, Danelli G, Evangelista M, Finco G, Gatti A, Gravino E, Launo C, Loreto M, Mediati R, Mokini Z, Mondello E, Palermo S, Paoletti F, Paolicchi A, Petrini F, Piacevoli Q, Rizza A, Sabato AF, Santangelo E, Troglio E, Mattia C (2010). SIAARTI study group. Postoperative pain treatment SIAARTI recommendations 2010. Short version. Minerva Anestesiol.

[13] Maddali P, Moisi M, Page J, Chamiraju P, Fisahn C, Oskouian R, Tubbs RS (2017). Anatomical complications of epidural anesthesia: a comprehensive review. Clin Anat.

[14] Guffey PJ, McKay WR, McKay RE (2010). Case report: epidural hematoma nine days after removal of a labor epidural catheter. Anesth Analg.

[15] Lagerkranser M, Lindquist C (2017). Neuraxial blocks and spinal haematoma: review of 166 cases published 1994 - 2015. Part 2: diagnosis, treatment, and outcome. Scand J Pain.

[16] Lawton MT, Porter RW, Heiserman JE, Jacobowitz R, Sonntag VKH, Dickman CA (1995). Surgical management of spinal epidural hematoma: relationship between surgical timing and neurological outcome. J Neurosurg.

